# Safety and efficacy of long-acting injectable cabotegravir as preexposure prophylaxis to prevent HIV acquisition

**DOI:** 10.1097/QAD.0000000000003494

**Published:** 2023-01-25

**Authors:** Virginia A. Fonner, Kathleen Ridgeway, Ariane van der Straten, Lara Lorenzetti, Nhi Dinh, Michelle Rodolph, Robin Schaefer, Heather-Marie A. Schmidt, Van Thi Thuy Nguyen, Mopo Radebe, Hortencia Peralta, Rachel Baggaley

**Affiliations:** a FHI 360 Global Health and Population Research, Durham, North Carolina; b ASTRA Consulting and Center for AIDS prevention Studies (CAPS), Department of Medicine, San Francisco, California, USA; c World Health Organization, Global HIV, Hepatitis and STIs Programmes, Geneva, Switzerland; d World Health Organization, Country Office, Hanoi, Viet Nam; e World Health Organization, Country Office, Pretoria, South Africa; f Pan American Health Organization, Washington, DC, USA; g UNAIDS Regional Office for Asia and the Pacific, Bangkok, Thailand.

**Keywords:** HIV prevention, long-acting injectable cabotegravir, preexposure prophylaxis, systematic review

## Abstract

HIV remains a significant burden, despite expanding HIV prevention tools. Long-acting injectable cabotegravir (CAB-LA) is a new preexposure prophylaxis (PrEP) product. We reviewed existing evidence to determine the efficacy and safety of CAB-LA as PrEP to inform global guidelines.

**Design:**

Systematic review and meta-analysis.

**Methods:**

We systematically reviewed electronic databases and conference abstracts for citations on CAB-LA from January 2010 to September 2021. Outcomes included HIV infection, adverse events, drug resistance, pregnancy-related adverse events, and sexual behavior. We calculated pooled effect estimates using random-effects meta-analysis and summarized other results narratively.

**Results:**

We identified 12 articles/abstracts representing four multisite randomized controlled trials. Study populations included cisgender men, cisgender women, and transgender women. The pooled relative risk of HIV acquisition comparing CAB-LA to oral PrEP within efficacy studies was 0.21 (95% confidence interval: 0.07–0.61), resulting in a 79% reduction in HIV risk. Rates of adverse events were similar across study groups. Of 19 HIV infections among those randomized to CAB-LA with results available, seven had integrase strand transfer inhibitor (INSTI) resistance. Data on pregnancy-related adverse events were sparse. No studies reported on sexual behavior.

**Conclusions:**

CAB-LA is highly efficacious for HIV prevention with few safety concerns. CAB-LA may lead to an increased risk of INSTI resistance among those who have acute HIV infection at initiation or become infected while taking CAB-LA. However, results are limited to controlled studies; more research is needed on real-world implementation. Additional data are needed on the safety of CAB-LA during pregnancy (for mothers and infants) and among populations not included in the trials.

## Background

Globally, approximately 37.7 million people were living with HIV and an additional 1.5 million people newly acquired HIV in 2020 [1]. Millions of people remain at risk of acquiring HIV despite an array of tools available to prevent acquisition, including preexposure prophylaxis (PrEP).

PrEP is the use of antiretroviral drugs by HIV-uninfected individuals to reduce the risk of HIV acquisition. In 2015, the World Health Organization (WHO) recommended oral PrEP containing tenofovir disoproxil fumarate (TDF) as a prevention choice for people at substantial risk of HIV as part of combination prevention approaches [2]. Daily oral PrEP containing TDF (and event-driven oral PrEP for people assigned male at birth [3]) was the only recommended form of PrEP until 2021 when WHO made a conditional recommendation for use of the dapivirine vaginal ring as PrEP among cisgender women at substantial risk of HIV acquisition [4]. Since then, potential PrEP drugs and delivery mechanisms have expanded [5].

Results from two randomized controlled trials (RCTs) of long-acting injectable cabotegravir (CAB-LA) as PrEP [HIV Prevention Trials Network (HPTN) 083 and 084] have become available [6,7], open-label extension studies are underway, and implementation projects are planned. Cabotegravir is an integrase strand transfer inhibitor (INSTI), which was first approved by the United States Food and Drug Administration (FDA) as an injectable form of HIV prevention [8] and treatment (CAB combined with rilpivirine) [9]. Potential benefits of CAB-LA compared to oral PrEP include its long-acting duration, increased user discretion, and lower user burden (i.e. long-acting bi-monthly provider-administered injection versus a pill taken daily or during times of risk). Programs implementing oral PrEP have noted challenges with uptake and effective use among oral PrEP users [10–14]. Furthermore, strong preferences for long-acting prevention products among certain populations, such as adolescent girls and young women, have been reported [15,16].

Modern contraceptive coverage increased as more options became available [17,18]. Similarly, expanding the PrEP toolkit by introducing options such as CAB-LA could increase coverage among those who could most benefit by allowing informed choice [19]. However, potential drawbacks must be considered. For example, the development of INSTI drug resistance is possible among those taking CAB-LA who develop HIV infection (or who had undetected HIV prior to initiation) [20]. This possibility is concerning given that it may confer cross-resistance to other INSTIs, such as dolutegravir, part of WHO-recommended first-line HIV treatment.

To inform WHO global guidelines regarding whether CAB-LA should be offered as a prevention choice for people at substantial risk of HIV infection, we conducted a systematic review and meta-analysis to synthesize evidence regarding the safety and efficacy of CAB-LA as PrEP.

## Methods

### Search strategy and selection criteria

We searched five electronic databases from January 1, 2010 through September 30, 2021: PubMed, CINAHL (Cumulative Index to Nursing and Allied Health Literature), Global Health, The Cochrane Central Register of Controlled Trials and Embase (search terms in Appendix S1). We also searched the International AIDS Conference, International AIDS Society Conference on HIV Pathogenesis, Treatment, and Prevention, Conference on Retroviruses and Opportunistic Infections, and Research for Prevention Conference websites through February 2022. We searched registries of ongoing clinical trials, including clinicaltrials.gov and the WHO International Clinical Trials Registry Platform. We conducted iterative secondary reference searching and contacted selected experts to identify additional articles (through May 2022). Preprints of eligible articles were initially included, although published versions were ultimately included as they became available.

Citations meeting the following criteria were included: RCT, open-label extension of a trial, or PrEP demonstration project evaluating use of CAB-LA to prevent HIV infection; measured one or more key outcomes, comparing those randomized to CAB-LA versus daily oral PrEP or versus nonuse of CAB-LA, and published in a peer-reviewed journal, presented at a scientific conference, or unpublished work (or work undergoing peer review). Key outcomes included: HIV infection, any adverse event (AE) (operationalized as an grade 2 or higher AE), any stage 3 or 4 AE (operationalized as any serious adverse event to achieve comparability across studies), drug resistance, and sexual and reproductive health outcomes, including effectiveness of hormonal contraception and gender affirming hormones, adverse pregnancy events, condom use, number of sexual partners, curable STIs incidence.

Review articles, editorials, and other articles without primary data were excluded. No restrictions based on intervention or language were used in the search. Studies pertaining to values and preferences of injectable PrEP were excluded from the present study but were flagged during the screening process and are included and summarized in a separate review [21].

Initial citation screening was conducted by one reviewer; retained citations were subsequently screened independently by two reviewers, with differences resolved through consensus. Data on study design, populations, intervention, and outcomes were extracted in duplicate for eligible citations. We assessed risk of bias using the Cochrane Collaboration's Risk of Bias Tool [22].

### Data analysis

When studies presented comparable results pertaining to a certain outcome, meta-analysis was conducted using random-effects models in STATA v16 [23]. Variability between studies was assessed using the *I*^2^ statistic and *Q* test. When meta-analysis was infeasible, results were summarized narratively. We followed the Preferred Reporting Items for Systematic Reviews and Meta-Analyses (PRISMA) guidelines throughout the review process [24]. The protocol was prospectively registered with Prospero (CRD42021290713).

## Results

**Fig. 1:**
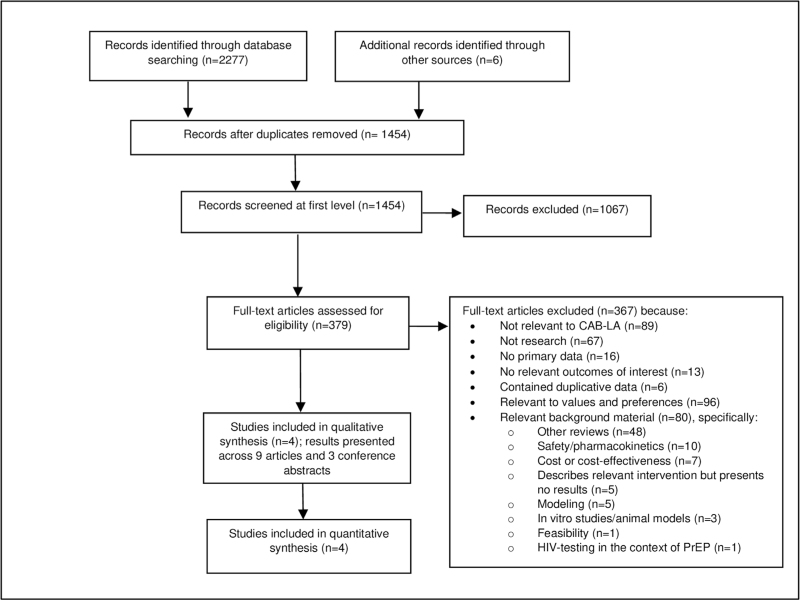
Study selection process.

Of 1450 unique citations identified, twelve eligible articles/abstracts containing data from four studies were included (Fig. 1). Two studies, HPTN 083 [6,25–27] and HPTN 084 [7,28,29], were phase 2b/3 trials assessing the efficacy of CAB-LA versus daily TDF-based oral PrEP (herein referred to as ‘efficacy studies’), and two studies, ÉCLAIR [30] and HPTN 077 [31–34], were phase 2a trials assessing CAB-LA safety and dosing (‘safety studies’). All studies were multisite RCTs, and risk of bias was low (Table S2, Supplemental Digital Content). HPTN 083 and HPTN 084 were stopped early for high safety and efficacy; following unblinding participants could receive access to open-label CAB-LA following protocol amendments [35,36]. We use the term ‘efficacy’ throughout our results given trials were designed to test CAB-LA under controlled circumstances. However, we recognize that ‘efficacy’ and ‘effectiveness’ exist on a continuum [37]. For example, some issues observed under controlled circumstances within PrEP trials, such as sub-optimal use, also reflect real-world conditions.

The figure demonstrates the disposition of citations during the searching and screening process. A search date of 2010 was selected to ensure not only studies pertaining to CAB-LA were included but also to ensure studies pertaining to values and preferences of injectable PrEP were included as this search simultaneously informed a related systematic review on this topic. Additionally, during the searching process, articles were retained as background material if they failed to meet the inclusion criteria but contained relevant information. Categories for background material included other reviews pertaining to PrEP, articles describing the safety and/or pharmacokinetics of CAB-LA, studies related to cost or cost-effectiveness of CAB-LA or injectable PrEP more generally, studies that described a relevant intervention but presented no results, such as published study protocols, modeling studies, studies related to CAB-LA using animal models, studies that described the feasibility of a relevant intervention without presenting results of interest, and articles related to HIV testing in the context of PrEP. Of the four studies included in the review, several articles were typically included per study, such as a main results paper, a paper containing laboratory findings, and secondary data analyses. Articles included in the qualitative synthesis had relevant results summarized narratively. Articles in the quantitative synthesis had key outcomes analyzed through random-effects meta-analysis. CAB-LA, long-acting injectable cabotegravir; PrEP, preexposure prophylaxis.

Across studies 8120 individuals were enrolled, with 4114 individuals randomized to receive CAB-LA (Table 1). HPTN 083 enrolled cisgender men and transgender women who have sex with men at-risk for HIV across 43 sites in the United States, Latin America, Africa, and Asia [6]. HPTN 084 enrolled cisgender women at-risk for HIV across seven sub-Saharan African countries [7]. ÉCLAIR enrolled men at low HIV risk in the United States [30], and HPTN 077 enrolled men and women at low HIV risk across Brazil, Malawi, South Africa, and the United States [33]. All participants were aged 18 years and above. No study sought to include people who inject drugs; pregnant and breastfeeding people were excluded. A small number of transgender men were included in HPTN 077 (*n* = 6) and HPTN 084 (*n* = 2) [7,33].

**Table 1:** Description of included studies.

Study	Study design	Location	Population	Sample size	Intervention description	Follow-up time
Phase 2b/3 studies (efficacy studies)						
HPTN 083	Multisite, double blind, double dummy, randomized controlled noninferiority trial	43 sites across Africa, Asia, Latin America, and the United States	Cisgender men who have sex with men and transgender women who have sex with men at-risk for HIV, aged ≥18 years	4570 (*n* = 2282 randomized to CAB-LA	Oral lead in phase: 5 weeks Injection phase: IM injection (gluteal muscle) of 600 mg of CAB or saline solution (every 8 weeks^a^) plus daily pill (placebo for intervention arm; TDF-FTC for placebo arm) Tail phase: All participants received daily oral open-label TDF-FTC; followed for 48 weeks	Median follow-up time: 1.4 years
HPTN 084	Multisite, double blind, double dummy, randomized controlled superiority trial	20 sites across 7 sub-Saharan African countries	Cisgender women at risk for HIV, 18−45 years old.	3224 (*n* = 1614 randomized to CAB-LA)		Median follow-up time: 1.24 years
Phase 2a studies (safety studies)						
ECLAIR	Multisite, double-blind randomized, placebo-controlled, phase 2a trial	10 sites in the United States	Men (defined as male sex at birth), HIV uninfected, and at low risk for HIV infection, 18–65 years old	*N* = 127 (*N* = 106 randomized to CAB-LA)	Oral lead in phase: 4-weeks, followed by 1 week washout period Injection phase: IM injection (800 mg of CAB or saline placebo) at 12-week intervals over 3 injection cycles. Tail phase: Followed up to 80 weeks	Primary safety outcomes measured at 41 weeks
HPTN 077	Multisite, double-blind randomized, placebo-controlled, phase 2a trial	8 sites in Brazil, Malawi, South Africa, and the United States.	All genders, HIV uninfected, and at low risk for HIV infection, 18–65 years old	*N* = 200 (*N* = 110 in cohort 1, including 82 randomized to CAB and *N* = 89 in cohort 2, including 69 randomized to CAB-LA)	Oral lead-in phase: Cohort 1 injection phase: 3 injections of CAB-LA 800 mg or saline placebo IM every 12 weeks for 3 injection cycles Cohort 2 injection phase: CAB-LA 600 mg or placebo IM for 5 injection cycles; the first 2 injections in Cohort 2 were separated by 4 weeks, the rest by 8 weeks. Tail phase: Followed up to 76 weeks after last injection	Primary safety outcomes measured at 41 weeks

CAB-LA, long-acting cabotegravir; IM, intramuscular; TDF-TFC, tenofovir disoproxil fumarate/emtricitabine.

a Cabotegravir is administered as a single 600-mg (3-mL) injection given 1 month apart for 2 consecutive months and continued with subsequent injections every 2 months thereafter. (https://www.accessdata.fda.gov/drugsatfda_docs/label/2021/215499s000lbl.pdf).

### HIV infection

**Fig. 2:**
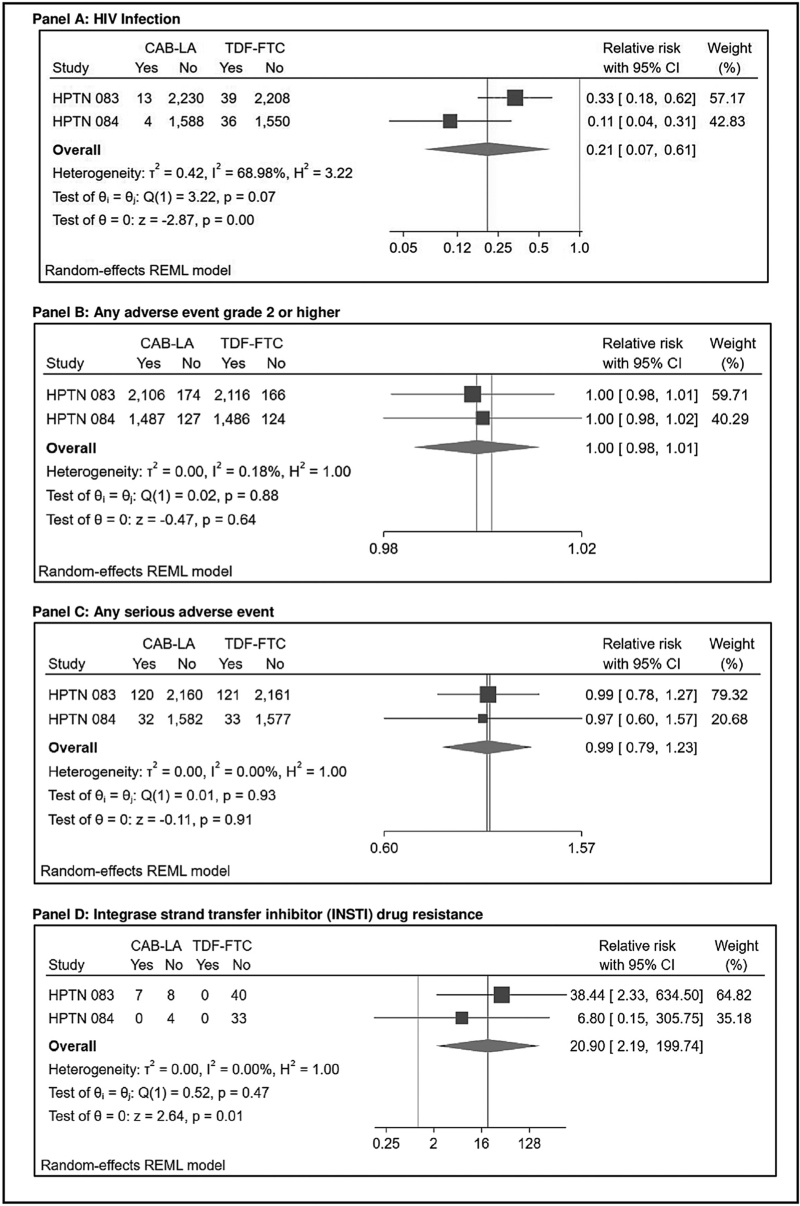
Forest plots for synthesized effect sizes within the efficacy studies.

Both efficacy studies reported on HIV infection as their primary outcome (Table S3, Supplemental Digital Content and Table S4, Supplemental Digital Content). Among 3857 individuals randomized to CAB-LA, 15 incident HIV infections occurred during the prespecified analysis period. Two additional infections (one each in HPTN 083 and HPTN 084) were originally classified as incident but later reclassified as baseline infections. Among 3857 individuals randomized to daily tenofovir/emtricitabine (TDF-FTC), 75 incident HIV infections occurred. The pooled relative risk (RR) of HIV infection comparing CAB-LA to oral PrEP was 0.21 [95% confidence interval (CI) 0.07−0.61, *P* = 0.004) (Fig. 2), corresponding to a 79% reduction in HIV risk. The imprecision surrounding this estimate relates to the relatively small number of HIV infections that occurred across studies. Additionally, given differences in populations and individual effect sizes, relatively high levels of heterogeneity were identified (*I*^2^ = 69.0, *Q* = 3.22). HPTN 083 and HPTN 084 categorized seroconversions based on timing of infection relative to study events (Table S5, Supplemental Digital Content). For HIV infections identified, retrospective testing of stored blood samples was conducted to estimate the timing of infection. In some cases, additional testing identified infections that were present at baseline but initially undetected.

The figure comprises four forest plots containing results from the random effects meta-analyses conducted among efficacy studies for four primary outcomes: HIV infection (Panel a), any adverse event grade 2 or higher (Panel b), any serious adverse event (Panel c), and integrase strand transfer inhibitor (INSTI) drug resistance (Panel d). Panel a shows the relative risk of HIV acquisition comparing CAB-LA to oral PrEP arms was 0.21 (95% CI: 0.07–0.61). Panel b shows the relative risk of experiencing any adverse event grade 2 or higher comparing CAB-LA to oral PrEP arms was 1.0 (95% CI: 0.98–1.01). Panel c shows the relative risk of experiencing any serious adverse event comparing CAB-LA to oral PrEP arms was 0.99 (95% CI: 0.79–1.23). Panel d shows the relative risk of having INSTI-resistance among HIV infections identified during the trials, comparing CAB-LA to oral PrEP arms, was 20.90 (95% CI: 2.19- 199.74). CAB-LA, long-acting injectable cabotegravir; CI, confidence interval; PrEP, preexposure prophylaxis.

Both efficacy studies reported a delay in HIV diagnosis among participants randomized to CAB-LA and oral PrEP. In HPTN 083, a delay in HIV detection was observed in 21/58 infections (36.8%), including in 11/16 infections among participants randomized to CAB-LA (68.8%) [27]. Among these infections, detection was delayed among all four baseline cases and seven incident cases. The mean delay for baseline cases within the CAB-LA arm was 62 days (range: 28–72) and 98 days (range: 35–185) for incident cases [27]. Within the oral PrEP arm, all three baseline cases had delayed detection as did 7/39 incident cases (17.9%). In HPTN 084, delays were identified in one and eight cases randomized to CAB-LA and oral PrEP, respectively [29]. The case randomized to CAB-LA had HIV infection present at baseline (identified through retrospective testing) but did not have HIV detected through clinic-based testing until week 33 (approximately 231 days) [29].

Three HIV infections were identified within safety studies. Among two infections within individuals randomized to CAB-LA (the third randomized to placebo), seroconversions occurred during the pharmacokinetic tail phase when CAB levels were below the quantifiable level of detection [30,33].

Recent HPTN 083 data, including one year of follow-up among participants following unblinding and the remaining period of the blinded portion that was excluded from the primary analysis reported above, demonstrated similar efficacy results [26]. In this extended period, 46 new incident HIV infections were detected, including 13 and 33 in the CAB-LA and oral PrEP groups, respectively. Two of the 13 additional HIV infections within the CAB-LA group were identified in the blinded portion of the study but after the per-protocol analysis period [26]. Notably, preliminary data from the unblinded portion of HPTN 083 demonstrated decreased use of oral PrEP and an increased number of missed or delayed visits to receive scheduled CAB-LA injection [26].

Regarding product use, injection coverage for HPTN 083 reached 91.5% of all person-years (defined as receiving an injection within <2 weeks delay of scheduled administration) [6]. In the TDF-FTC arm of HPTN 083, 74.2% of participants had tenofovir concentrations consistent with daily dosing (≥40 ng/ml) and over 86% had detectable tenofovir (≥0.31 ng/ml). In HPTN 084, injection coverage was 93% of all person-years [7]. In a random subset of those randomized to TDF-FTC (*n* = 405), 42% had tenofovir concentrations consistent with daily dosing and 56% had detectable tenofovir [7].

### Adverse events

We did not identify significant differences in any AE grade 2 or higher across study arms in efficacy studies [pooled risk ratio (RR) = 1.0, 95% CI 0.98–1.01, *P* = 0.64; Table S3, Supplemental Digital Content and Table S6, Supplemental Digital Content] or during the injection phases across arms within safety studies (pooled RR = 1.25, 95% CI 0.78–1.99, *P* = 0.35). However, investigators from ÉCLAIR noted a significant difference in AEs comparing CAB-LA to placebo [30], with injection site pain being the most commonly reported AE among those randomized to CAB-LA [27]. Notably, CAB-LA dosage in ÉCLAIR was higher than in Cohort 2 of HPTN 077 and in HPTN 083 and 084. Results of the synthesized effect for this outcome demonstrate substantial heterogeneity (*I*^2^ = 75.1, *Q* = 4.02), likely due to CAB-LA dosing differences across studies.

Within HPTN 083 and 084, proportions of participants reporting serious adverse events (SAEs) were similar across groups (pooled RR = 0.99, 95% CI 0.79–1.23, *P* = 0.91) [6,7] (Fig. 2, Table S3, Supplemental Digital Content and Table S7, Supplemental Digital Content). Similarly, within safety studies, there was no difference in SAEs comparing CAB-LA to placebo (pooled RR = 0.32, 95% CI 0.04–2.42, *P* = 0.27). All studies found that adverse events stemming from injection site reactions occurred more frequently among those randomized to CAB-LA (Figure S8, Supplemental Digital Content). Most reported reactions were mild to moderate in severity, and rates declined with subsequent injections.

HPTN 077, HPTN 083, and HPTN 084 reported on weight changes. Both HPTN 083 and 084 found annualized weight increases across study arms, with a statistically higher, albeit modest, annualized weight gain among those randomized to CAB-LA versus oral PrEP [6,7]. In HPTN 083 differences in weight change between arms were mostly observed during the first 40 weeks. In HPTN 084 there was an initial, immediate weight gain among participants randomized to CAB-LA [7], then gains of approximately 2 kg/year thereafter across both arms [7]. HPTN 077 found no difference in weight changes across arms and no differences in distributions of weight changes comparing sex at birth, body mass index (BMI), and other demographic characteristics [34].

### Drug resistance

Within the CAB-LA arms across HPTN 083 and 084, 20 HIV infections were identified during the per-protocol analysis period, including five baseline and 15 incident infections (Table S5, Supplemental Digital Content), of which 19 had drug resistance results available. INSTI resistance mutations were found in seven cases − all in HPTN 083 − resulting in a pooled relative risk of 20.90 (95% CI 2.19–199.74, *P* = 0.008) comparing CAB-LA to oral PrEP. The imprecision surrounding this estimate results from the few absolute numbers of INSTI-resistant cases identified. Of note, all resistant cases were identified following recent CAB exposure; no resistance was identified in infections occurring within the pharmacokinetic tail (Table S10, Supplemental Digital Content) [25,27,29]; however, data are limited as both HPTN 083 and 084 were stopped early and drug resistance results from the unblinded phases are not yet available. Phenotyping results among some resistant cases (*n* = 3) found varying susceptibility to commonly used integrase inhibitors; two of three cases were resistant to CAB [27].

### Pregnancy-related adverse events, hormone-related associations, and sexual behavior

Within studies including cisgender women (HPTN 077 and HPTN 084), participants were required to use effective contraception, thus data on pregnancy-related outcomes were limited. In HPTN 077, three pregnancies were identified, including one among a woman randomized to CAB-LA [33] (Table S11, Supplemental Digital Content). In HPTN 084, 49 confirmed pregnancies occurred among 48 participants, with no differences in pregnancy incidence by study arm [28]. Women randomized to CAB-LA experienced more pregnancy-related AEs (*n* = 6), although no AEs were considered product-related [28]. No congenital abnormalities were observed. The terminal phase half-life of CAB-LA appeared similar comparing pregnant women in HPTN 084 to nonpregnant women in HPTN 077 [28].

A secondary analysis from HPTN 084 analyzing the impact of hormonal contraceptives on CAB-LA pharmacokinetics [31] found that women on oral contraceptives had lower peak CAB concentrations compared to women not on hormonal contraception; however, no differences were observed across pharmacokinetic parameters when hormonal contraception was evaluated in aggregate [31]. This analysis did not assess the potential reverse drug-drug interaction (i.e. impact of CAB-LA on hormonal contraception) [31].

No study reported on the potential drug-drug interactions of gender affirming hormone therapy and CAB-LA. No studies reported on sexual behavior. HPTN 083 and 084 reported on incident rates of curable STIs, with no differences reported across arms [6,7] (Table S12, Supplemental Digital Content). HPTN 077 reported seven incident cases of STIs over study duration without disaggregation by study arm [33].

## Discussion

Our review suggests that CAB-LA is safe and highly effective for HIV prevention across studied populations and settings, demonstrating a 79% reduction in HIV risk compared to TDF-based daily oral PrEP. This estimate should be interpreted keeping in mind the heterogeneity of study populations and that relatively few HIV infections were identified. Although ethical considerations prevented trials from using a placebo comparator, a modeling study estimated that the efficacy of CAB-LA versus placebo was 93–95% among women [38].

No major safety concerns were identified. Mild injection site reactions appear to be frequent − with some variation across populations − although reaction frequency decreased over time. Data suggests unclear results regarding the association of CAB-LA and weight gain, as HTPN 077 found no difference in weight gain between arms but both HPTN 083 and 084 found a modest annualized weight gain among those randomized to CAB-LA versus oral PrEP. More research is warranted given these conflicting findings.

Drug resistance is a potential issue for CAB-LA implementation; however, low numbers of HIV infections among those randomized to CAB-LA resulted in even lower numbers of infections with identified INSTI resistance. More data from implementation studies are needed regarding the extent and implications of CAB-related drug resistance that occur but are not detected either before starting CAB-LA, during use, and after discontinuation during the pharmacokinetic tail phase.

Recent analyses demonstrate that screening for HIV infection before and during CAB-LA use with HIV RNA technology could potentially reduce INSTI-resistance by identifying cases of acute HIV earlier [25]. However, the impact of using RNA testing on INSTI resistance is unclear; such assays require more resources and access would vary by setting, potentially limiting CAB-LA implementation. There is likely some cross-resistance to dolutegravir and other integrase inhibitors, although the extent is unclear given the limited number of drug resistant cases [39]. Of note, WHO guidelines state that HIV testing for CAB-LA can be done using national testing algorithms composed of serology assays [40].

More implementation research is needed to determine optimal HIV testing strategies in the context of CAB-LA. Additionally, delays in HIV diagnosis reported among those exposed to CAB-LA needs further study as it is unclear whether a delay in diagnosis contributes to the emergence of drug resistance and the potential onward transmission of resistant strains. Results from included studies confirm prior investigations suggesting exposure to antiretroviral drugs, including through oral PrEP, during acute infection can suppress viral replication, thus delaying diagnosis in some instances [41,42]. However, more data are needed on the duration of delays by PrEP product, characteristics associated with delays, and associations of delays with emerging drug resistance.

This review highlights several potentially important implementation considerations as CAB-LA is now recommended by WHO as an additional HIV prevention option [40]. First, several incident HIV infections occurred during the oral lead-in phase. Since injectable PrEP might be a necessity for people who cannot sustain oral PrEP use, it is critical to weigh the risks (e.g., HIV infection) and benefits (e.g. tolerability assurance) of including an oral lead-in phase within injectable PrEP programs. The U.S. FDA approved the use of CAB-LA as PrEP without a required oral lead-in phase [8]. Secondly, recent data from the unblinded portion of HPTN 083 suggest a declining coverage of person-time protected by CAB-LA than seen within the blinded portion [22]. Results of the unblinded portion of HPTN 084 were recently presented, with similar efficacy to the blinded portion, although results on coverage compared to the blinded portion were not reported [43].

Our results highlight that people receiving CAB-LA had a lower risk of HIV acquisition than those receiving oral TDF-FTC. However, we are unable to disentangle whether this result is driven by differing efficacies between the agents themselves, or by the disparate routes of administration and subsequent differences in adherence burdens, or both. Although CAB-LA has a lower burden of user-adherence than oral PrEP, it is critical to promote persistence by ensuring timely follow-up clinical visits for subsequent injections, by making them convenient, accessible, integrated into on-going clinical services (where applicable), and supported by culturally competent, unbiased providers. People who discontinue CAB-LA must be aware of ways to prevent HIV infection during the pharmacokinetic tail (e.g. dapivirine vaginal ring, oral PrEP, condoms, and/or postexposure prophylaxis). Given that inability or unwillingness to use oral pills might drive preferences for CAB-LA, identifying suitable, effective prevention alternatives following CAB-LA discontinuation is key [44–46].

Additionally, questions remain about variability within the pharmacokinetic properties of CAB-LA among diverse populations and individuals. For example, results from HPTN 077 found that CAB-LA had a significantly longer terminal phase half-life in participants assigned female sex at birth, as compared to those assigned male sex at birth [32]. HPTN 077 also found that CAB-LA had a longer terminal phase half-life among participants with an above median BMI, as compared to participants with a below median BMI [32]. An additional study from HPTN 077 found that genetic variants of enzymes that metabolize CAB-LA exist and could affect the rate at which CAB-LA is metabolized [47]. A better understanding of variability of CAB-LA's pharmacokinetics could improve optimization of dosing tailored to different populations.

More research is needed regarding CAB-LA among populations not well represented in trials, including youth aged <18 years, sex workers, transgender men and other gender diverse people, people who use drugs, and pregnant and breastfeeding people. Safety and acceptability data will soon be available from two trials conducted among adolescents (HPTN 083-01 and HPTN 084-01). Additionally, data from the HPTN 084 open-label extension study are expected from women who chose to continue taking CAB-LA during pregnancy.

This review was conducted using rigorous systematic review methods and involved a broad search to ensure comprehensiveness. However, it is possible that studies were missed. The ability to synthesize effects across studies using meta-analysis was limited due to the small number of comparable studies. Due to differences in comparators, drug dosing, and study duration, results from efficacy studies were not combined with results from safety studies.

Based on evidence identified, CAB-LA was found to be a safe, highly efficacious form of PrEP. However, since results were focused on controlled studies, additional research is needed to understand implementation of CAB-LA in real-world settings and across populations, particularly those not represented in the trials. More research is also needed on outcomes with sparse data, such as drug resistance among those exposed to CAB-LA and safety during pregnancy and breastfeeding (for mothers and infants), as well as aspects of CAB-LA program implementation, such as identifying optimal HIV testing algorithms, strategies to promote adherence to injections, and integration of CAB-LA into existing services.

## Acknowledgements

The study was conceptualized by R.B., M.R., R.S., V.F., and L.L. V.F. and K.R. drafted the study protocol with input from A.V.S., L.L., and N.D. V.F., K.R., L.L., and N.D. conducted the screening of citations, with AVS helping to resolve differences in eligibility determination. V.F. and K.R. abstracted and analyzed data. V.F. drafted the initial manuscript, with all co-authors, including M.P., H.P., and V.T.T.N., provided substantial contributions to subsequent iterations. All co-authors have reviewed and approve of the final version.

We sincerely appreciate the help of Allison Burns of FHI 360 who conducted the literature searches. We also sincerely appreciate the review and feedback from the WHO Guideline Development Group, and other reviewers. We are also grateful for participants of the trials which were included in this review.

Funding: This work was made possible by the generous support of the American people through the U.S. President's Emergency Plan for AIDS Relief (PEPFAR) and the U.S. Agency for International Development (USAID) cooperative agreements 7200AA19CA00002 and 7200AA21CA00011. The contents are the responsibility of the EpiC and MOSAIC projects and do not necessarily reflect the views of PEPFAR, USAID, or the U.S. Government. WHO staff time was supported by grants from Unitaid and the Bill & Melinda Gates Foundation. Unitaid, and the Bill & Melinda Gates Foundation have awarded grants to the World Health Organization to enable this study. The authors alone are responsible for the views expressed in this article and they do not necessarily represent the views, decisions or policies of the institutions with which they are affiliated.

### Conflicts of interest

There are no conflicts of interest.

## Supplementary Material

Supplemental Digital Content
