# Supplementary material for: Safety and efficacy of long-acting injectable cabotegravir as preexposure prophylaxis to prevent HIV acquisition
Source: AIDS. 2023 Jan 25;37(6):957–66. doi: 10.1097/QAD.0000000000003494 (PMC10090368; doi:10.1097/QAD.0000000000003494)
Supplement: Supplemental Digital Content [file aids-37-0957-s001.docx]

# Supplementary Data File

## S1: Search terms for electronic database search

**PubMed**

(Cabotegravir OR "CAB-LA" OR injectable OR injection OR injections[Mesh] OR "long-acting" OR GSK1265744 OR "sustained release" OR "extended release" OR delayed-action preparations[Mesh] OR “controlled release” OR “slow release” OR “timed release” OR “prolonged-action” OR “prolonged action”) AND (“pre-exposure prophylaxis” OR “primary prevention” OR “PrEP” OR “HIV seronegativity” OR “HIV uninfected”) AND (hiv[MeSH] or "hiv infections"[MeSH] or "acquired immunodeficiency syndrome"[MeSH])

Limited to 2010-2021

**CINAHL, Global Health**

(Cabotegravir OR “CAB-LA” OR injectable OR injection OR injections OR “long-acting” OR GSK1265744 OR “sustained release” OR “extended release” OR “controlled-release” OR “slow release” OR “timed release” OR “prolonged-action” OR “prolonged action”)

AND

(“pre-exposure prophylaxis” OR “primary prevention” OR “PrEP” OR “HIV seronegativity” OR “HIV uninfected”)

AND

(hiv OR “hiv infections” OR “acquired immunodeficiency syndrome”)

Limited to 2010 – 2021

**Embase**

(Cabotegravir OR “CAB-LA” OR injectable OR injection OR injections OR “long-acting” OR GSK1265744 OR “sustained release” OR “extended release” OR “controlled-release” OR “slow release” OR “timed release” OR “prolonged-action” OR “prolonged action” OR “delayed-action” OR “delayed-action preparations”) AND (“pre-exposure prophylaxis” OR “primary prevention” OR “PrEP” OR “HIV seronegativity” OR “HIV uninfected”) AND (hiv OR “hiv infections” OR “acquired immunodeficiency syndrome”)

Limited to Embase, 2010-2021

**Cochrane**

(Cabotegravir OR “CAB-LA” OR injectable OR injection OR injections OR “long-acting” OR GSK1265744 OR “sustained release” OR “extended release” OR “controlled-release” OR “slow release” OR “timed release” OR “prolonged-action” OR “prolonged action”) in all text AND (“pre-exposure prophylaxis” OR “primary prevention” OR “PrEP” OR “HIV seronegativity” OR “HIV uninfected”) in all text AND (hiv or “hiv infections” or “acquired immunodeficiency syndrome”) in all text

Limited to 2010-2021

## S2. Risk of bias for included studies

|  | Random sequence generation (selection bias) | Allocation concealment  (selection bias) | Blinding of participants and personnel (performance bias) | Blinding of outcome assessment (detection bias) | Incomplete outcome data addressed (attrition bias) | Selective reporting (reporting bias) | Other bias |
| --- | --- | --- | --- | --- | --- | --- | --- |
| HPTN 083 | Low risk | Low risk | Low risk | Low risk | Low risk | Low risk | Low risk |
| HPTN 084 | Low risk | Low risk | Low risk | Low risk | Low risk | Low risk | Low risk |
| ÉCLAIR | Low risk | Low risk | Low risk | Low risk | Unclear risk^a^ | Unclear risk^a^ | Low risk |
| HPTN 077 | Low risk | Low risk | Low risk | Low risk | High risk^b^ | Low risk | Low risk |

^a^ Unclear risk due to lack of a publicly available protocol.

^b^ High risk of attrition bias based on <80% retention within cohort 1 across both arms.

## S3. Summary of synthesized effects of key outcomes

| **Outcome** | **No. studies**  **(Study names)** | **Risk ratio** | **95% CI** | ***P* value** | **I^2^** | **Q** |
| --- | --- | --- | --- | --- | --- | --- |
| HIV infection | 2 (HPTN 083, 084) | 0.21 | (0.07 – 0.61) | 0.004 | 69.0 | 3.22 |
| Any adverse event |  |  |  |  |  |  |
| Efficacy studies | 2 (HPTN 083, 084) | 1.00 | (0.98 – 1.01) | 0.64 | 0.18 | 0.02 |
| Safety studies | 2 (ÉCLAIR, HPTN 077) | 1.25 | (0.78 - 1.99) | 0.35 | 75.1 | 4.02 |
| Any serious adverse event |  |  |  |  |  |  |
| Efficacy studies | 2 (HPTN 083, 084) | 0.99 | (0.79 – 1.23) | 0.93 | 0.0 | 0.01 |
| Safety studies | 2 (ÉCLAIR, HPTN 077) | 0.32 | (0.04 - 2.42) | 0.27 | 32.9 | 1.49 |
| INSTI drug resistance | 2 (HPTN 083, 084) | 20.90 | (2.19 - 199.74) | 0.008 | 0.00 | 0.52 |

*I^2^ and Q are tests for heterogeneity within meta-analysis. INSTI refers to integrase strand transfer inhibitors, the drug class for cabotegravir.*

## S4. HIV infection outcomes across efficacy studies

| **Study** | **HIV incidence rate (per 100 person years) among groups exposed to cabotegravir (95% CI)** | **HIV incidence rate (per 100 person years) among groups exposed to TDF-FTC (95% CI)** | **Hazard ratio**  **(95% CI)** | **P value** | **Corresponding relative reduction in risk** |
| --- | --- | --- | --- | --- | --- |
| **HPTN 083** | 0.41^a, b, c^ | 1.22^b^ | 0.34 (0.1 8 - 0.62) | <0.001 | 66% |
| **HPTN 084** | 0.20 (0.06 - 0.52)^d^ | 1.85 (1.3 - 2.57) | 0.12 (0.05 - 0.31)^e^ | <0.0001 | 88% |
| **HPTN 083 (open-label extension)** | 0.76^b^ | 2.20^b^ | 0.33 (0.17- 0.66) | Not reported | 67% |

^a^ Estimate includes one HIV infection in the CAB-LA arm that was initially classified as an incident infection but was re-adjudicated as a baseline infection after further analysis. Taking this case into account, the adjusted HIV incidence rate was 0.37 (95% CI: 0.16 – 0.58) and the corresponding hazard ratio was 0.32 (95% CI: 0.16 – 0.58).

^b^95% confidence intervals for the HIV incidence rates were not reported.

^c^ Estimate includes pre-specified analysis. One HIV Infection in the CAB-LA arm that was initially classified as an incident infection but was re-adjudicated as a baseline infection after further analysis. When taking this case into account, the adjusted HIV incidence rate was 0.15 (95% CI: 0.03 – 0.45) and the corresponding hazard ratio was 0.09 (95% CI: 0.04 – 0.27). Additionally, post hoc analyses in HPTN 083 identified 2 additional HIV infections within the CAB-LA arm and 2 additional infections within the TDF-FTC arm. When taken into account, the revised HIV incidence rate among those randomized to CAB-LA is 0.44 per 100 person years and the revised HIV incidence rate among those randomized to TDF-FTC is 1.29 per 100 person-years. The revised hazard ratio is 0.34 (0.18–0.62), which is very similar to the hazard ratio presented in the pre-specified analysis included in the table above.

^d^ Estimate includes pre-specified analysis. One HIV Infection in the CAB arm that was initially classified as an incident infection but was re-adjudicated as a baseline infection after further analysis. When taking this case into account, the CAB-LA group incidence was recalculated as 0.15 per 100 person-years (95% CI: 0.03 to 0.45). The revised hazard ratio (HR 0.09, 95% CI 0.04 to 0.27); p-value <0.0001), demonstrating a 91% relative reduction in HIV risk.

^e^ Adjusted for site and group-sequential design

## S5. HIV infections identified within groups randomized to CAB-LA by timing of infection

| **Timing of infection** | **HPTN 083^a^**  (n=16 infections in CAB-LA arm) | **HPTN 084**  (n=4 infections in CAB-LA arm) | **Total**  (n=20 infections) | **Total infections with INSTI resistance identified out of total within category, %** |
| --- | --- | --- | --- | --- |
| **Baseline infection** | 4 | 1 | 5 | 1/5 (20%) |
| **Infection following no recent CAB exposure** | 5 | 2 | 7 | 0^a^ (0%) |
| **Infection during the oral lead-in phase** | 3 | 0 | 3 | 2/3 (67%) |
| **Breakthrough infection** | 4^b^ | 1^c^ | 5 | 4/5 (80%) |

*CAB-LA:* long-acting cabotegravir*; INSTI:* integrase strand transfer inhibitor

^a^ Numbers in the table reflect all infections identified during the blinded, pre-specified analyses period. Two additional infections (classified as breakthrough infections) occurred during the blinded portion of HPTN 083 but following the period pre-specified for analysis. Eleven infections were identified among the CAB-LA group during the unblinded portion, including one breakthrough infection, three with delayed CAB injections (i.e., missed the scheduled injection for a period ranging from 1 to 3.6 months), and seven occurred >6 months after the last CAB injection. No drug resistance information was available on these cases at the time of this report.

^b^ One participant in HPTN 083 randomized to CAB-LA who became infected with HIV following no recent CAB exposure did not have results available from resistance testing; therefore, it is possible that one of the seven cases that occurred following no recent CAB exposure had some INSTI resistance.

^c^ Infection occurred in a participant with delayed injection visits (her last injection occurred 16.1 weeks prior to her first HIV positive visit)

## S6. Any adverse event (AE)^a^ outcomes across included studies

| **Study** | **No. participants reporting any AE/participants randomized to CAB-LA (%)** | **No. participants reporting any AE/participants randomized to TDF-FTC or placebo (%)** |
| --- | --- | --- |
| HPTN 083 | 2106/2280 (92.4%) | 2216/2282 (92.7%) |
| HPTN 084 | 1487/1614 (92%) | 1486/1610 (92%) |
| ÉCLAIR^b^ | 75/94 (71%) | 10/21 (48%) |
| HPTN 077^c^ | 122/134 (91%) | 38/43 (88%) |

*CAB-LA:* long-acting cabotegravir*; AE:*  adverse event*; TDF-FTC:* tenofovir disoproxil fumarate/emtricitabine

^a^Defined as any grade 2 or higher adverse event or higher

^b^ Includes AEs experienced during the injection phase. Authors report difference in grade 2 or higher AE were statistically significant (p>0.005). In addition to reporting any grade 2 or higher AE, ÉCLAIR also reported the proportion of study participants experiencing any AE (Grades 1-4), which included 92/94 (98%) of participants experiencing any AE in the CAB-LA arm and 19/21 (90%) experiencing any AE in the placebo arm during the injection phase.

^c^ Includes grade 2 or higher AEs that occurred during the injection phase of the study.

## S7. Serious adverse events (SAEs) across included studies

| **Study** | **No. participants reporting SAE/participants randomized to active CAB-LA** | **No. participants reporting SAE/participants randomized to TDF-FTC or placebo** |
| --- | --- | --- |
| HPTN 083^a^ | 120/2280 (5.3%) | 121/2282 (5.3%) |
| HPTN 084^b^ | 32/1614 (2%) | 33/1610 (2%) |
| ÉCLAIR^c^ | 0/121 (0%) | 1/21 (5%) |
| HPTN 077 | 4/134 (3%) | 2/43 (4.7%) |

*CAB-LA:* long-acting cabotegravir*; SAE:* serious adverse event*; TDF-FTC:* tenofovir disoproxil fumarate/emtricitabine

^a^ Study also reported number and proportion of any grade 3 or 4 adverse events: 727/2280 (32%) in the CAB-LA arm and 767/2282 (33%) in the TDF-TFC arm.

^b^ Study also reported number and proportion of grade 3 or higher AE: 276/1614 (17%) in the CAB-LA arm and 280/1610 (17%) in the TDF-FTC arm

^b^ Study also reported the number and proportion of any drug-related grade 3 or 4 adverse events occurring during the injection phase of the study: 19/94 (20%) in the CAB-LA arm and 0/21 in the placebo arm.

## S8. Injection site reactions by study arm and study

*Figure legend.* The orange bars represent the percentage of participants reporting injection site reactions (ISRs) among arms receiving active CAB-LA injections. The blue bars represent the arms receiving placebo injections. In HPTN 083, 81.5% of participants randomized to CAB-LA who received one or more injections reported at least one ISR [6]. Within the TDF-FTC arm (with placebo injections), 31.3% experienced ISRs. In HPTN 084, 38% of participants in the CAB-LA arm experienced any ISR as compared to 11% in the TDF-FTC arm [7]. In HPTN 077, 38% of participants randomized to CAB-LA experienced an ISR versus 2% in the placebo group. There were no reported differences in ISR rates across males and females receiving CAB-LA [23]. [7, 23]. In ÉCLAIR, 93% of participants randomized to CAB-LA reported ISRs compared with 57% of participants randomized to placebo [20].

## S9. Drug resistance identified across efficacy studies

|  | **HPTN 083** | | **HPTN 084** | |
| --- | --- | --- | --- | --- |
| Resistance | CAB-LA arm (n=16 HIV infections) | TDF-FTC arm (n=42 HIV infections) | CAB-LA arm  (n=4 HIV infections) | TDF-FTC arm (N=36 HIV infections) |
| NNRTI | 2 | 7 | 0 | 8 |
| NNRTI/NRTI | 1 | 3 | 0 | 1 |
| NRTI | 0 | 1 | 0 | 0 |
| INSTI | 6 | 0 | 0 | 0 |
| INSTI/NNRTI | 1 | 0 | 0 | 0 |

*CAB-LA:* long-acting cabotegravir*; INSTI:* integrase strand transfer inhibitor*; NNRTI:* Non-nucleoside reverse transcriptase inhibitors; *NRTI:* *Nucleoside reverse transcriptase inhibitors; TDF-FTC:* tenofovir disoproxil fumarate/emtricitabine

## S10. INSTI drug resistance outcomes across efficacy studies

| **Study** | **Number of INSTI resistant strains^a^/ number of HIV infections (including baseline infections) among those randomized to CAB-LA (%)** | **Number of INSTI resistant strains^a^/number of HIV infections (including baseline infections) randomized to TDF-FTC (%)** |
| --- | --- | --- |
| **HPTN 083** | 7^b^/15^c^ (47%) | 0/40^c^ (0%) |
| **HPTN 084** | 0/4 (0%) | 0/33^c^(0%) |

^a^ Defined as major INSTI mutation. Minor mutations and polymorphisms are not included in this total. In the TDF-FTC arm of HPTN 083, there was one case with minor INSTI mutation. In HTPN 084, one HIV case randomized to the CAB-LA arm had an INSTI L741 polymorphism. In the TDF-FTC arm of HPTN 084, there were 10 INSTI polymorphisms identified but none had INSTI resistance.

^b^ The cases of INSTI resistance were identified in HPTN 083 among 1 participant with a baseline infection, 2 participants during the oral lead-in phase, and 4 participants with a breakthrough infection.

^c^ Denominator reflects the total number of HIV cases (incident and baseline infections) that were successfully tested for drug resistance. In HPTN 083, three cases in the CAB-LA were initially not included (one case had failed analysis and two had no viremic visit). Similarly, in the TDF-FTC arm, two cases were excluded (two had no viremic visits). However, data were recently made available (presented at CROI 2022) on further genotypic testing on two cases within the CAB-LA arm in which initial testing was infeasible due to low viremia. Both cases had INSTI resistance. In HPTN 084, all HIV infections within the CAB-LA had drug resistance testing performed.

## S11. Pregnancy incidence and pregnancy-related outcomes across studies that included cisgender women

| **Study** | **Pregnancy incidence among women randomized to CAB-LA per 100 PY**  **(95% CI)** | **Pregnancy incidence among women randomized to TDF-FTC or placebo per 100 PY (95% CI)** | **No. of pregnancies** | **Adverse pregnancy outcomes observed** |
| --- | --- | --- | --- | --- |
| HPTN 084 | 1.5 (1.0 - 2.2) | 1.0 (0.6, 1.6) | 29 in CAB arm; 20 in TDF-FTC arm^a^ | - Known pregnancy outcomes in 31/49 cases - In CAB arm: 13 live births, 1 pregnancy loss at 20-36 weeks, and 3 losses at <20 weeks (including elective terminations), no known congenital abnormalities in 11 cases (unknown in 3 additional cases) - In TDF-FTC arm: 10 live births, 2 pregnancy losses at 20-36 weeks, and 1 loss <20 weeks (including elective terminations); no known congenital abnormalities in 12 cases (unknown in 1 additional case) - No congenital abnormalities identified - CAB-LA participants (n=6) experienced more pregnancy-related AE, including premature rupture of membranes (n=2), pre-eclampsia (n=1), oligohydramnios (n=1), incomplete abortion (n=1) and morning sickness (n=1) than TDF/FTC participants (morning sickness n=1). None of these AE were considered as product-related - Incidence of grade 2 or higher AE comparing women who received at least one injection and became pregnant: 113/100 py (95% CI: 69.3-185.4/100 py) in the CAB-LA arm vs. 166/100 py (95% CI: 102.2-271.0/100 py) in the TDF/FTC arm (p=0.064) |
|  |  |  |  |  |
| HPTN 077 | NR | NR | 1 in CAB arm (during tail phase) and 2 in placebo arm | - In CAB-LA arm: 1 pregnancy, resulting in live birth at 38 weeks, no birth defects - In placebo arm: 2 pregnancies. One resulted in a miscarriage at 11 weeks (likely due to infection with the Zika virus) and one resulted in a live birth with no apparent birth defects |
|  |  |  |  |  |

*AE:* adverse event; *CAB-LA:* long-acting cabotegravir*; py:* person years; *NR*: not reported; *TDF-FTC:* tenofovir disoproxil fumarate/emtricitabine

## S12. Incidence of curable STIs over study duration within included studies

| **Study** | **STI outcome as reported in studies** | **STI incidence over the course of the study in cabotegravir arm^2^** | **STI incidence over the course of the study in TDF-FTC arm (for efficacy studies) or placebo arm (for safety studies)^2^** |
| --- | --- | --- | --- |
| HPTN 083 | Chlamydia (rectal and urine), Gonorrhea (rectal and urine), and syphilis | - Syphilis, rate per 100 PY: 16.6 - Gonorrhea (urine), rate per 100 PY: 2.77 - Gonorrhea (rectal), rate per 100 PY: 11.1 - Chlamydia (urine), rate per 100 PY: 4.44 - Chlamydia (rectal), rate per 100 PY: 15.8 | - Syphilis (rate per 100 PY): 16.7 - Gonorrhea (urine), rate per 100 PY: 2.13 - Gonorrhea (rectal), rate per 100 PY: 11 - Chlamydia (urine), rate per 100 PY: 4.67 - Chlamydia (rectal), rate per 100 PY: 17.8 |
| HPTN 084 | Chlamydia, Trichomoniasis, and Gonorrhea^a^ | - Chlamydia: 261/1614 (16%) - Trichomoniasis: 124/1614 (8%) | - Chlamydia: 287/1610 (18%) - Trichomoniasis: 109/1610 (7%) |
| HPTN 077 | Chlamydia (rectal and urine), Gonorrhea (rectal and urine), and syphilis | NR^b^ | NR^b^ |

*py:*  person years; NR: *not reported*

^a^ Overall STI incidences were also reported (not disaggregated by study arm): incidence of chlamydia: 19.6 per 100 py (95% CI 18 to 21); incidence of gonorrhea: 7.7 per 100 py (95% CI 6.8 to 8.7). The incidence of STIs did not vary significantly by study group (p value or result from statistical test not reported)

^b^ Seven incident infections reported among 6 participants overall, although results are not disaggregated by arm: rectal chlamydia (2 cases), urinary chlamydia (1 case), urinary gonorrhea (1 case), dual rectal and urinary gonorrhea (1 case), and early syphilis (1 case). All STIs except for syphilis were in female participants.
